# A Sweetpotato Auxin Response Factor Gene (*IbARF5*) Is Involved in Carotenoid Biosynthesis and Salt and Drought Tolerance in Transgenic *Arabidopsis*

**DOI:** 10.3389/fpls.2018.01307

**Published:** 2018-09-11

**Authors:** Chen Kang, Shaozhen He, Hong Zhai, Ruijie Li, Ning Zhao, Qingchang Liu

**Affiliations:** ^1^Key Laboratory of Sweetpotato Biology and Biotechnology, Ministry of Agriculture, Beijing Key Laboratory of Crop Genetic Improvement, Laboratory of Crop Heterosis and Utilization, Ministry of Education, College of Agronomy and Biotechnology, China Agricultural University, Beijing, China; ^2^College of Agronomy, Qingdao Agricultural University, Qingdao, China

**Keywords:** sweetpotato, *IbARF5*, *Arabidopsis*, carotenoid content, salt and drought tolerance

## Abstract

Auxin response factors (ARFs) compose a family of transcription factors and have been found to play major roles in the process of plant growth and development. However, their roles in plant carotenoid biosynthesis and responses to abiotic stresses are rarely known to date. In the present study, we found that the *IbARF5* gene from sweetpotato (*Ipomoea batatas* (L.) Lam.) line HVB-3 increased the contents of carotenoids and enhanced the tolerance to salt and drought in transgenic *Arabidopsis*. The transgenic *Arabidopsis* plants exhibited the increased abscisic acid (ABA) and proline contents and superoxide dismutase (SOD) activity and the decreased H_2_O_2_ content. Furthermore, it was found that *IbARF5* positively regulated the genes associated with carotenoid and ABA biosynthesis and abiotic stress responses. These results suggest that *IbARF5* is involved in carotenoid biosynthesis and salt and drought tolerance in transgenic *Arabidopsis*. This study provides a novel *ARF* gene for improving carotenoid contents and salt and drought tolerance of sweetpotato and other plants.

## Introduction

In nature, more than 750 kinds of carotenoids are characterized structurally, which are widely found in bacteria, fungi, algae, and plants (Hirschberg, 2001; Takaichi, 2011). The biosynthesis pathway of carotenoids has been extensively studied in plants, and nearly all of the key genes have been isolated and characterized (Cunningham and Gantt, 1998; Fraser and Bramley, 2004; Colasuonno et al., 2017; Kang et al., 2018). Abiotic stresses, especially salt and drought, seriously affect the productivity and cultivation expansion of crop plants worldwide, accordingly, to develop their high tolerance to salt and drought is highly desirable (Zhu, 2002; Lindemose et al., 2013; Zhai et al., 2016; Li et al., 2017). As the precursor of abscisic acid (ABA), carotenoids have functional roles in development and environmental adaptation of plants (Schwartz et al., 2003; Nambara and Marion-Poll, 2005; Mehrotra et al., 2014; Li, 2015; Moreno et al., 2016). Thus, increasing the contents of carotenoids helps to enhance the adaptation of plants to harsh environments.

Auxin response factors (ARFs) constitute a family of plant specific transcription factors. A typical ARF protein contains a B3-DNA binding domain in the highly conserved N-terminal region (Ulmasov et al., 1997; Hagen and Guilfoyle, 2002; Mei et al., 2018). ARFs mediate responses to auxin and have been shown to be implicated in senescence (Ellis et al., 2005), hormone signaling (Li et al., 2006) and developmental programs (Krogan et al., 2012). In rice, *OsARF1* was auxin-regulated and classified as a primary auxin responsive gene (Waller et al., 2002). In *Arabidopsis, ARF2* mediated ABA response (Wang et al., 2011); *MP/ARF5* regulated embryo and flower patterning and vascular differentiation (Hardtke and Berleth, 1998; Krogan et al., 2012); *ARF6* and *ARF8* promoted jasmonic acid production and flower maturation (Nagpal et al., 2005); *NPH4/ARF7* and *ARF19* controlled leaf expansion and lateral root growth (Okushima et al., 2005; Wilmoth et al., 2005). In tomato, *SlARF2* regulated lateral root formation and flower senescence (Ren et al., 2017); *ARF4* controlled sugar metabolism (Sagar et al., 2013); *ARF10* increased chlorophyll and sugar accumulation during fruit development (Mei et al., 2018); *ARF5* regulated fruit set and development (Liu et al., 2018). However, the roles of ARFs in plant carotenoid biosynthesis and abiotic stress responses are rarely known to date.

Sweetpotato (*Ipomoea batatas* (L.) Lam.) is an important food crop worldwide, which provides rich carbohydrates and carotenoids for human consumption (Teow et al., 2007; Zhai et al., 2016). This crop can also be used for bioenergy production on marginal lands due to its high adaption to harsh environments (Liu et al., 2014). Sweetpotato breeders are focusing on improving carotenoid contents and abiotic stresses tolerance of this crop. Kang et al. (2017) summarized the improvement of carotenoids by gene engineering in sweetpotato. Overexpression of the genes related to carotenoid biosynthesis have been shown to increase the contents of carotenoids and enhance the tolerance to abiotic stresses in sweetpotato (Kim et al., 2012, Kim et al., 2013b; Yu et al., 2013; Kim et al., 2014; Li et al., 2017; Kang et al., 2018). To date, ARFs have not been reported in sweetpotato. In this study, we found that the *IbARF5* gene from storage roots of sweetpotato is involved in carotenoid biosynthesis and salt and drought tolerance in transgenic *Arabidopsis*.

## Materials and Methods

### Plant Materials

Sweetpotato line HVB-3 with high carotenoid content was employed to clone the *IbARF5* gene in this study. The expressed sequence tag (EST) for *IbARF5* was obtained from the transcriptome data of HVB-3 developed by Li et al. (2015). *Arabidopsis* wild type (Columbia-0, WT) was used for characterizing the *IbARF5* gene.

### Isolation and Sequence Analysis of *IbARF5*

Total RNA was extracted from freshly harvested storage roots of HVB-3 and transcribed into first-strand cDNA according to the method of Kang et al. (2018). The full-length cDNA of *IbARF5* was amplified with specific primers (**Supplementary Table S1**) by rapid amplification of cDNA ends (RACE) method. Genomic DNA isolated from *in vitro*-grown plants of HVB-3 was used to amplify the genomic sequence of *IbARF5*. The *IbARF5* cDNA was analyzed by an online BLAST^1^. The open-reading frame (ORF) Finder^2^ was used to predict the ORF of *IbARF5*. The DNAMAN software was applied to align the amino acid sequence of IbARF5 with those of ARF proteins from different plant species. The MEGA 7.0 software was employed to conduct the phylogenetic analysis with the neighbor-joining (NJ) method. Exon–intron structure was constructed using Splign tool^3^. The molecular weight and theoretical isoelectric point (*p*I) of IbARF5 were calculated at http://web.expasy.org/compute_pi/.

### Subcellular Localization of IbARF5

The *IbARF5* ORF amplified with specific primers (**Supplementary Table S1**) was ligated into pMDC83. pMDC83-*IbARF5*-*GFP* and pMDC83-*GFP* (as control) were transiently expressed in the onion epidermal cells with a GeneGun (HeliosTM, Biorad, United States). After co-cultivation on Murashige and Skoog (MS) medium (pH 5.8) at 28°C for 24 h, the onion cells were examined under a laser scanning confocal fluorescence microscope (Nikon Inc., Melville, NY, United States).

### Transactivation Activity Assay of IbARF5 in Yeast

Transactivation activity of IbARF5 in yeast (*Saccharomyces cerevisiae*) was assayed as described by Jiang et al. (2014). The corresponding regions of *IbARF5* were PCR-amplified using specific primers (**Supplementary Table S1**) and integrated into the yeast expression vector pGBKT7 (pBD). Expression vectors pBD-*IbARF5*, pBD-*IbARF5-1*, pBD-*IbARF5-2*, pBD-*IbARF5-3*, pGAL4 (as positive vector), and pBD (as negative vector) were transferred into the yeast strain AH109, respectively. The transactivation activity was determined as described in the yeast protocols handbook (PT3024-1; Clontech, Mountain View, CA, United States).

### Expression Analysis of *IbARF5* in Sweetpotato

Total RNA was isolated from storage root, stem, and leaf tissues of the 100-day-old HVB-3 and used to analyze the expression of *IbARF5* by quantitative real-time PCR (qRT-PCR) with its specific primers (**Supplementary Table S1**). *Ibactin* (AY905538) was served as an internal control. Comparative *C*_T_ method was employed to quantify the gene expression (Schmittgen and Livak, 2008).

After cultured on MS medium for 4 weeks, the HVB-3 plants were treated in liquid MS media containing H_2_O (as control), 200 mM NaCl, 20% PEG6000 and 100 μM ABA, respectively, and sampled at 0, 2, 4, 6, 12, 24, and 48 h after treatment for analyzing the expression of *IbARF5*.

### Production of the Transgenic *Arabidopsis* Plants

The overexpression vector pC3301-121-*IbARF5* was constructed through inserting *35S-IbARF5-NOS* into pCAMBIA3301. The recombinant vector was introduced into the *Agrobacterium tumefaciens* strain GV3101. The dipping flower method was applied to transform *Arabidopsis* and putatively transgenic *Arabidopsis* seeds were sown on MS medium with 12.5 mg L^-1^ phosphinothricin (PPT) for selecting transgenic plants. Histochemical GUS assay (Jefferson et al., 1987) and PCR analysis were used to identify the transgenic *Arabidopsis* plants. Transgenic *Arabidopsis* was planted in pots with a soil, vermiculite and humus mixture (1:1:1, v/v/v) to obtain T_3_ seeds.

### Measurement of Carotenoid Contents

Leaves (2-week-old) and seeds of the transgenic *Arabidopsis* plants were applied to extract α-carotene, lutein, β-carotene, β-cryptoxanthin, and zeaxanthin. High performance liquid chromatography (HPLC) system was used to determine their contents (Li et al., 2017).

### Assay for Salt and Drought Tolerance

One-week-old *in vitro*-grown seedlings of transgenic *Arabidopsis* and WT were treated on MS media with 200 mM NaCl and 300 mM mannitol, respectively. After 2 weeks, their root length and fresh weight (FW) were investigated. Furthermore, the transgenic and WT seedlings were planted for 2 weeks in pots with a soil, vermiculite and humus mixture (1:1:1, v/v/v) and subsequently irrigated with a 33 mL of 300 mM NaCl solution for each pot once every 2 days for 2 weeks, or stressed by drought for 4 weeks followed by 2 days re-watering. The transgenic plants and WT grown for 6 weeks under normal condition were used as control. The proline and H_2_O_2_ contents and superoxide dismutase (SOD) activity in the transgenic plants and WT grown in pots for 4 weeks under normal condition, 1 week under 300 mM NaCl stress after 2 weeks of normal treatment, and 2 weeks under drought stress after 2 weeks of normal treatment, respectively, were determined with Assay Kits (Comin Biotechnology Co., Ltd. Suzhou, China). The ABA content was measured as described by Gao et al. (2011). Twenty-seven plants in three pots with nine plants per pot were treated for each line.

For ABA sensitivity assay, the transgenic and WT seeds were sown on MS media with 0, 0.5, and 1 μM ABA, respectively. After 1 week, their germination and cotyledon opening and greening rates were investigated. Fifty seeds of each line on a plate were analyzed.

### Expression Analysis of the Related Genes

Leaves (2-week-old) and seeds of the transgenic *Arabidopsis* plants and WT were applied to analyze the expression of the key genes in carotenoid biosynthesis. The leaves of the transgenic plants and WT potted for 4 weeks under normal condition, 1 week under 300 mM NaCl stress after 2 weeks of normal treatment, and 2 weeks under drought stress after 2 weeks of normal treatment, respectively, were used for analyzing the expression of the genes associated with ABA biosynthesis and abiotic stress responses. The specific primers of *Atactin* (NM112764) as internal control and the related genes were listed in **Supplementary Table S1**.

### Statistical Analysis

All experiments were conducted with three biological replicates. Data presented as the mean ± SE were analyzed with Student’s *t*-test (two-tailed analysis) at *P* < 0.05 and *P* < 0.01.

## Results

### Cloning and Sequence Analysis of *IbARF5*

**FIGURE 1:**
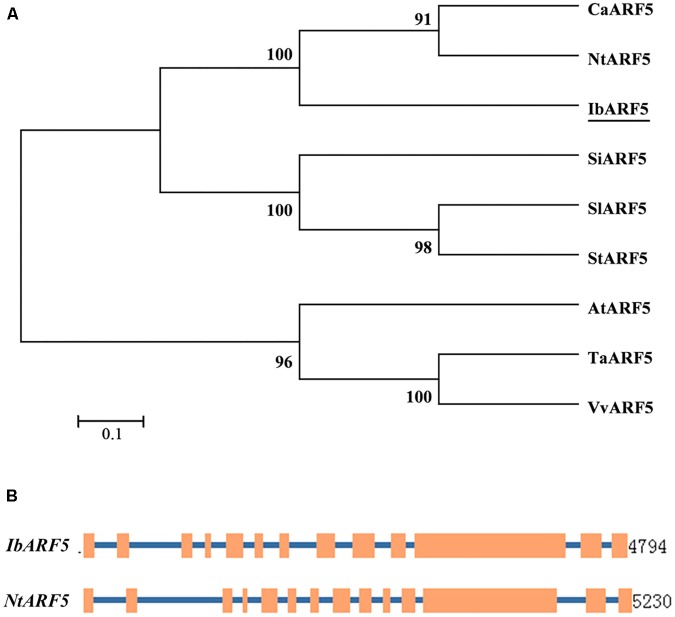
Phylogenetic tree of ARF5 in different plant species **(A)** and comparison of exon and intron constituents between *IbARF5* and *NtARF5* **(B)**. Exons are represented by boxes and introns by lines.

The 3757-bp cDNA of the *IbARF5* gene contained a 2841-bp ORF encoding a 946-aa polypeptide with a molecular weight of 104.84 kDa and a predicted *p*I of 5.17. The IbARF5 protein shared a high sequence identity with ARF5 proteins in *Nicotiana tabacum* (XP_016465083.1, 74%), *Capsicum annuum* (XP_016568464.1, 72%), *Sesamum indicum* (XP_011083507.1, 72%), *Solanum lycopersicum* (NP_001234545.1, 72%), *Solanum* *tuberosum* (XP_006342026.1, 72%) and *Vitis vinifera* (XP_003634382.2, 68%). It contained one plant-specific B3-DNA binding domain, one Auxin_resp and one AUX_IAA (**Supplementary Figure S1**). Phylogenetic analysis showed that IbARF5 had a close relationship with that of *N. tabacum* (**Figure 1A**). The 4794-bp genomic DNA of *IbARF5* contained 13 exons and 12 introns (**Figure 1B**).

### IbARF5 Is Localized to Nuclei

**FIGURE 2:**
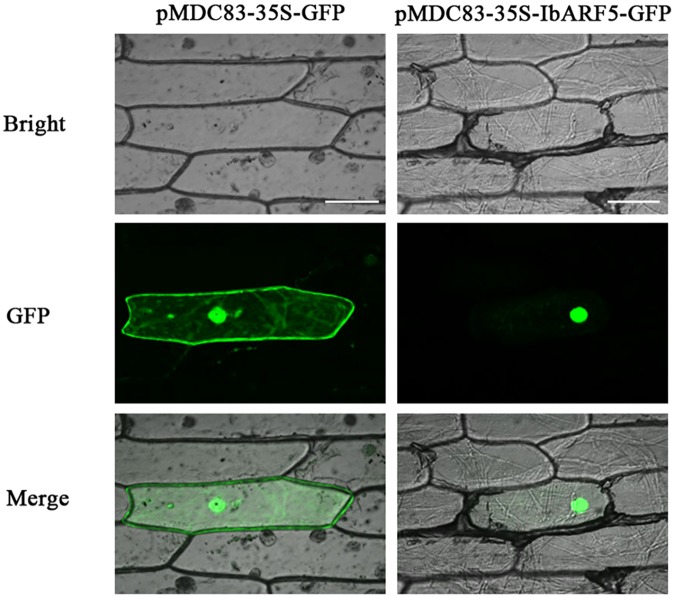
Subcellular localization of IbARF5 in onion leaf hypodermal cells. Confocal scanning microscopic images show localizations of IbARF5-GFP fusion proteins to nuclei in the right column vs. GFP as control in the left column. Bars = 20 μm.

The images from onion epidermal cells indicated that the green fluorescence emitted by IbARF5-GFP was exclusively distributed over the nuclei of the cells (**Figure 2**). These results showed that IbARF5 was localized to nuclei.

### IbARF5 Shows Transactivation Activity in Yeast

**FIGURE 3:**
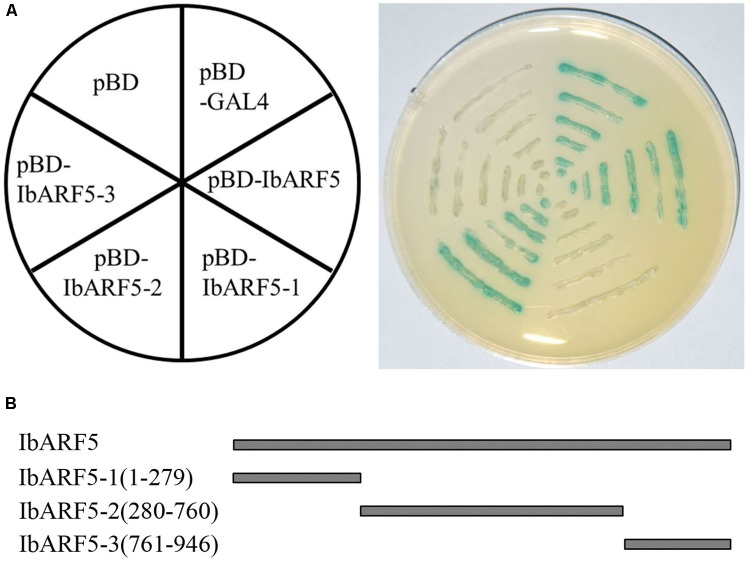
Transactivation activity of IbARF5 in the yeast. **(A)** the pBD-GAL4 vector as positive control; pBD-IbARF5; pBD-IbARF5-1; pBD-IbARF5-2; pBD-IbARF5-3; the empty pBD vector as negative control. The culture solution of the transformed yeast was drawn onto SD plate without tryptophan and histidine. **(B)** Different portions of IbARF5.

The yeast two-hybrid system was applied to identify a possible transactivation activity of IbARF5. The yeast cells harboring pBD-GAL4, pGBKT7-*IbARF5* and pGBKT7-*IbARF5*-2, respectively, grew well on synthetic dropout (SD) plate without tryptophan and histidine and exhibited β-galactosidase activity, but the cells bearing pBD, pGBKT7-*IbARF5*-1, and pGBKT7-*IbARF5*-3, respectively, failed to grow (**Figure 3A**). These results demonstrated that IbARF5 might act as a transcription activator, and its transactivation activity was determined by the middle region, IbARF5-2 (**Figure 3B**).

### Expression Patterns of *IbARF5* in Sweetpotato

**FIGURE 4:**
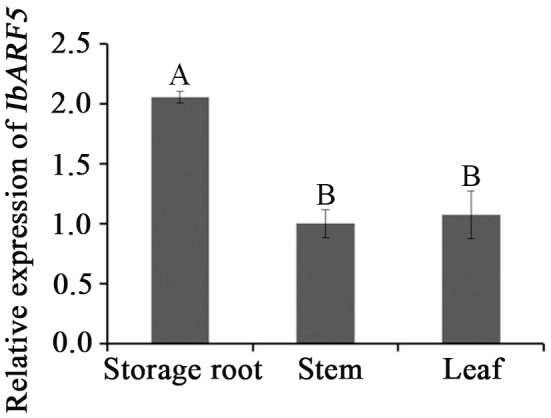
Expression analysis of *IbARF5* in storage root, stem, and leaf tissues of HVB-3. Data are presented as means ± SE (*n* = 3). Different capital letters indicate a significant difference at *P* < 0.01 by Student’s *t*-test.

**FIGURE 5:**
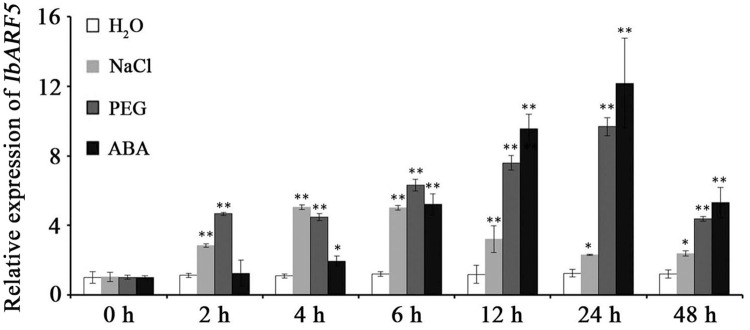
Expression analysis of *IbARF5* in the *in vitro*-grown plants of HVB-3 after different times (h) in response to H_2_O, 200 mM NaCl, 20% PEG6000 and 100 μM ABA, respectively. Data are presented as means ± SE (*n* = 3). ^∗^ and ^∗∗^ indicate a significant difference from that of WT at *P* < 0.05 and *P* < 0.01, respectively, by Student’s *t*-test.

Quantitative real-time PCR analysis revealed that *IbARF5* exhibited higher expression level in the storage roots of HVB-3 than in its leaves and stems (**Figure 4**). Its expression in HVB-3 was strongly induced by NaCl, PEG6000 and ABA, and peaked (5.03-fold) at 4 h, (9.68-fold) at 24 h, and (12.18-fold) at 24 h, respectively (**Figure 5**).

### Production of the Transgenic *Arabidopsis* Plants

Putatively transgenic *Arabidopsis* seeds formed the plants on MS medium with 12.5 mg L^-1^ PPT. GUS assay and PCR analysis confirmed that 6 of the randomly sampled 60 plants were transgenic plants, named L1, L2, …, L6, respectively, from which T_3_ were generated. *IbARF5* showed high expression levels in the transgenic *Arabidopsis* plants, especially L1, L4, L5, and L6 (**Supplementary Figure S2**).

### *IbARF5* Increases Carotenoid Contents

The lutein and zeaxanthin contents were significantly increased, but α-carotene and β-carotene contents were not changed and β-cryptoxanthin was not detected in leaves of L1, L4, L5, and L6 (**Table 1**). In seeds of these four transgenic plants, the lutein, β-carotene, and zeaxanthin contents were significantly increased, but α-carotene was not detected and β-cryptoxanthin content was significantly increased only in L5 and L6 (**Table 2**). The total carotenoid contents in leaves and seeds were increased by 1.13–1.16 folds and 1.32–1.78 folds, respectively (**Tables 1** and **2**).

**Table 1:** Carotenoid contents in leaves of the *IbARF5*-overexpressing *Arabidopsis* plants.

	Carotenoids content (μg g^-1^ FW)					
Lines	α-carotene	Lutein	β-carotene	β-cryptoxanthin	Zeaxanthin	Total
WT	0.149 ± 0.005	13.275 ± 0.581	7.601 ± 0.467	n.d.	0.044 ± 0.010	21.070 ± 1.020
L1	0.166 ± 0.011	17.475 ± 0.694^∗∗^	6.751 ± 0.552	n.d.	0.119 ± 0.007^∗∗^	24.511 ± 1.038^∗∗^
L4	0.151 ± 0.004	16.750 ± 1.221^∗∗^	6.849 ± 0.330	n.d.	0.147 ± 0.016^∗∗^	23.896 ± 0.949^∗^
L5	0.145 ± 0.006	16.775 ± 0.315^∗∗^	6.657 ± 0.755	n.d.	0.147 ± 0.004^∗∗^	23.993 ± 0.854^∗^
L6	0.153 ± 0.007	17.532 ± 1.210^∗∗^	6.717 ± 0.111	n.d.	0.126 ± 0.009^∗∗^	24.529 ± 1.109^∗∗^

*Leaves from 2-week-old *Arabidopsis* plants were sampled for the quantification of carotenoids. FW, fresh weight; n.d., not detectable. Data are presented as mean ± SE (*n* = 3). ^∗^ and ^∗∗^ indicate a significant difference from that of WT at *P* < 0.05 and *P* < 0.01, respectively, by Student’s *t*-test. *t*-test*.

**Table 2:** Carotenoid contents in seeds of the *IbARF5*-overexpressing *Arabidopsis* plants.

	Carotenoids content (μg g^-1^ DW)					
Lines	α-carotene	Lutein	β-carotene	β-cryptoxanthin	Zeaxanthin	Total
WT	n.d.	1.470 ± 0.041	0.178 ± 0.003	0.020 ± 0.005	0.089 ± 0.009	1.755 ± 0.048
L1	n.d.	1.907 ± 0.028^∗∗^	0.270 ± 0.031^∗^	0.023 ± 0.006	0.119 ± 0.010^∗∗^	2.318 ± 0.051^∗∗^
L4	n.d.	2.519 ± 0.207^∗∗^	0.262 ± 0.035^∗^	0.038 ± 0.006	0.153 ± 0.008^∗∗^	2.972 ± 0.241^∗∗^
L5	n.d.	2.625 ± 0.150^∗∗^	0.309 ± 0.057^∗∗^	0.046 ± 0.002^∗^	0.143 ± 0.005^∗∗^	3.123 ± 0.194^∗∗^
L6	n.d.	2.303 ± 0.287^∗∗^	0.386 ± 0.059^∗∗^	0.052 ± 0.003^∗∗^	0.144 ± 0.023^∗∗^	2.885 ± 0.313^∗∗^

*Seeds from Arabidopsis plants were harvested for the quantification of carotenoids. DW, dry weight; n.d., not detectable. Data are presented as mean ± SE (*n* = 3). ^∗^ and ^∗∗^ indicate a significant difference from that of WT at *P* < 0.05 and *P* < 0.01, respectively, by Student’s *t*-test. *t*-test*.

### *IbARF5* Enhances Salt and Drought Tolerance

**FIGURE 6:**
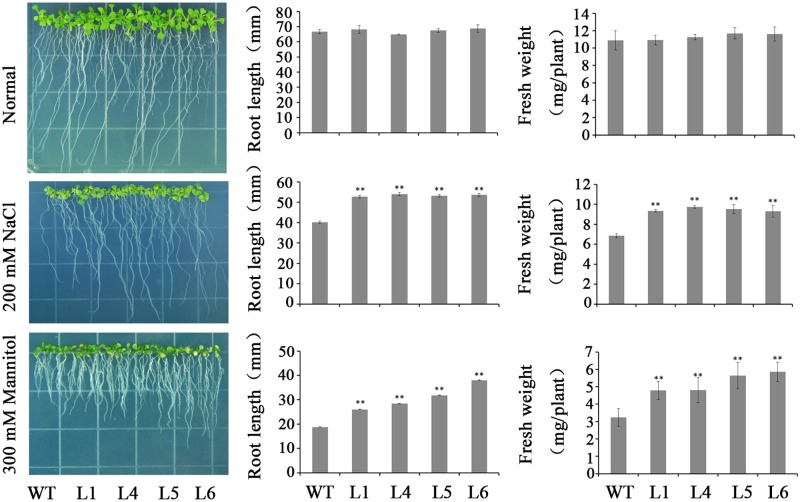
Responses of the transgenic *Arabidopsis* seedlings and WT cultured for 2 weeks on MS medium with 200 mM NaCl and 300 mM mannitol, respectively. Data are presented as mean ± SE (*n* = 3). ^∗∗^ indicates a significant difference from that of WT at *P* < 0.01 by Student’s *t*-test.

Four transgenic *Arabidopsis* plants, L1, L4, L5, and L6, and WT seedlings showed no significant differences in rooting and FW on MS medium without stresses (**Figure 6**). However, the transgenic plants exhibited good rooting and increased FW in contrast to WT on MS media with 200 mM NaCl and 300 mM mannitol, respectively (**Figure 6**).

**FIGURE 7:**
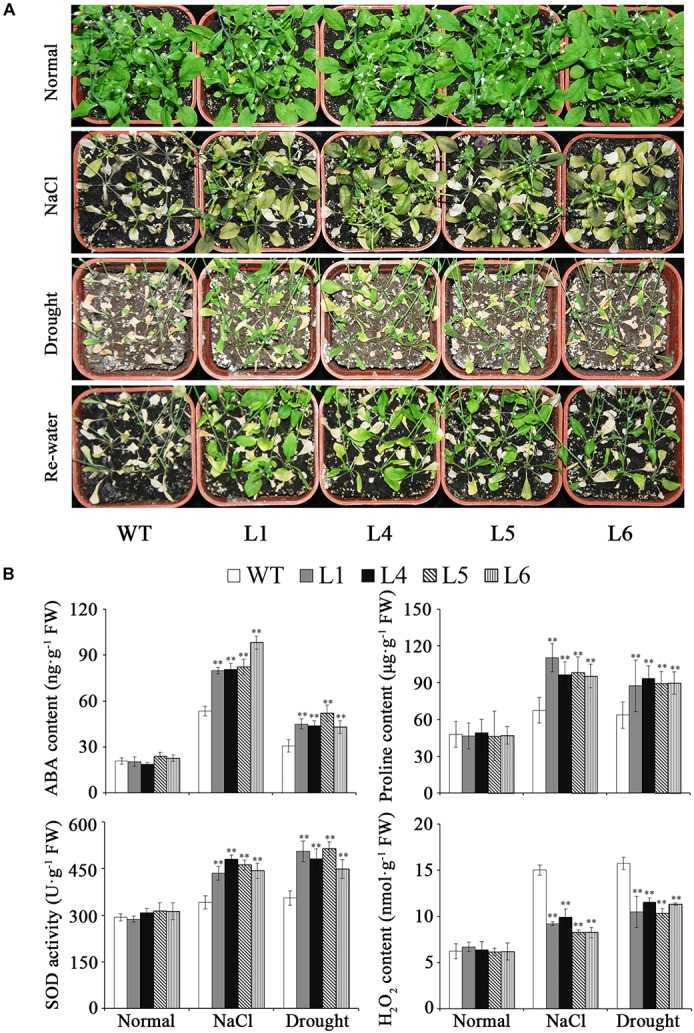
Responses of the transgenic *Arabidopsis* plants and WT grown in pots under salt and drought stresses. **(A)** Phenotypes of transgenic plants vs. WT grown for 6 weeks under normal condition, 2 weeks under 300 mM NaCl stress after 2 weeks of normal treatment, and 4 weeks under drought stress followed by 2 days re-watering after 2 weeks of normal treatment, respectively. **(B)** ABA, proline and H_2_O_2_ contents and SOD activity in the transgenic plants and WT grown for 4 weeks under normal condition, 1 week under 300 mM NaCl stress after 2 weeks of normal treatment, and 2 weeks under drought stress after 2 weeks of normal treatment, respectively. Data are presented as mean ± SE (*n* = 3). ^∗∗^ indicates a significant difference from that of WT at *P* < 0.01 by Student’s *t*-test.

The transgenic plants and WT grown in pots showed similar growth trends under normal conditions (**Figure 7A**). Under NaCl and drought stresses, the transgenic plants showed good growth, while WT almost died (**Figure 7A**). Furthermore, it was found that the ABA and proline contents were increased, SOD activity was enhanced and H_2_O_2_ content was decreased in the transgenic plants (**Figure 7B**).

**FIGURE 8:**
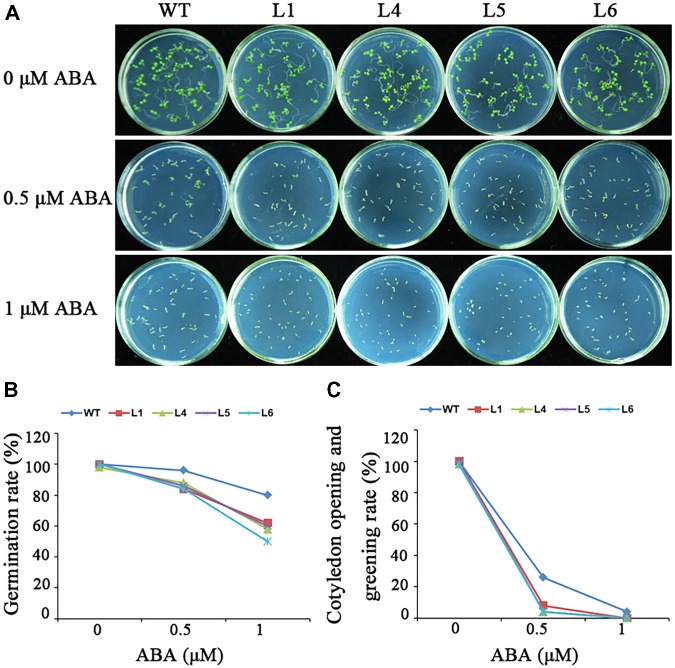
Responses of the transgenic *Arabidopsis* and WT seeds sown on MS medium with 0, 0.5, and 1 μM ABA for 1 week. **(A)** Growth vigor of the transgenic and WT seedlings. **(B)** Germination rates of the transgenic and WT seeds. **(C)** Cotyledon opening and greening rates of the transgenic and WT seeds.

No obvious differences in seed germination were observed between the transgenic plants and WT under normal condition (**Figure 8**). Under the treatment with different ABA concentrations, the germination of the transgenic seeds was more sensitive to ABA-elicited inhibition than that of WT though both germination rate and cotyledon opening and greening rate of the transgenic and WT seeds declined (**Figure 8**). These results demonstrated that *IbARF5* might participate in the ABA signaling pathway.

### *IbARF5* Up-Regulates the Genes Involved in Carotenoid and ABA Biosynthesis and Abiotic Stress Responses

**FIGURE 9:**
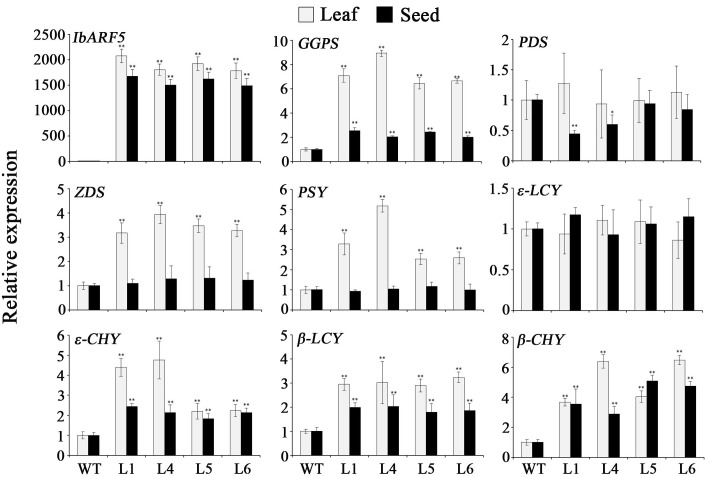
Transcript levels of *IbARF5* and carotenoid biosynthesis genes in leaves and seeds of the transgenic *Arabidopsis* plants and WT. Data are presented as means ± SE (*n* = 3). ^∗^ and ^∗∗^ indicate a significant difference from that of WT at *P* < 0.05 and *P* < 0.01, respectively, by Student’s *t*-test.

**FIGURE 10:**
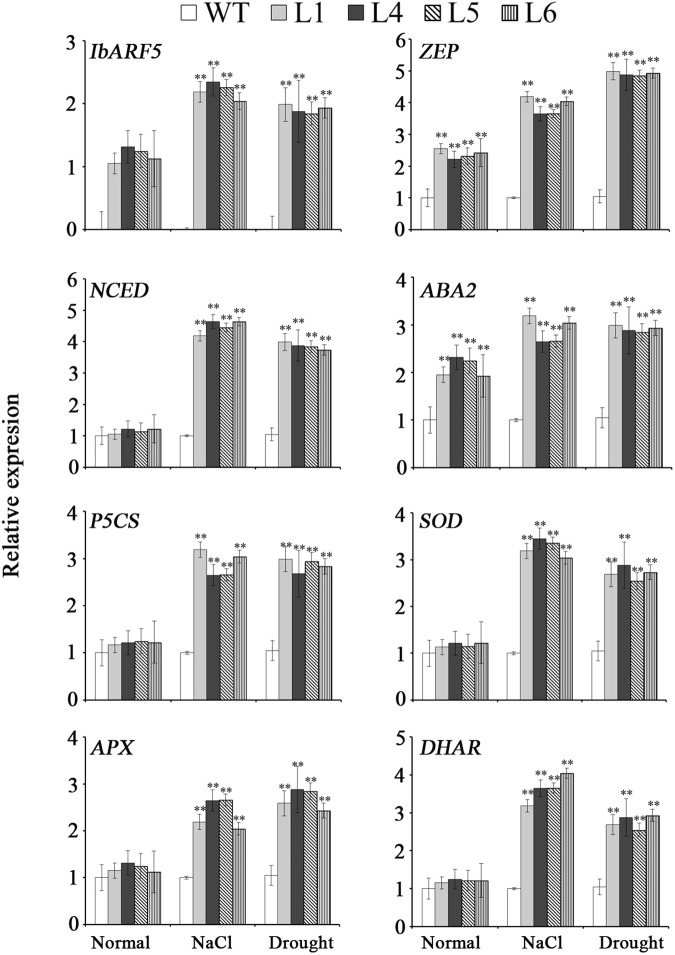
Transcript levels of salt and drought responsive genes in leaves of transgenic *Arabidopsis* plants and WT pot-grown for 4 weeks under normal condition, 1 week under 300 mM NaCl stress after 2 weeks of normal treatment, and 2 weeks under drought stress after 2 weeks of normal treatment, respectively. Data are presented as mean ± SE (*n* = 3). ^∗∗^ indicates a significant difference from that of WT at *P* < 0.01 by Student’s *t*-test.

The genes encoding the key enzymes in carotenoid biosynthesis, eranylgeranyl pyrophosphate (GGPS), ζ-carotene desaturase (ZDS), phytoene synthase (PSY), 𝜀-carotene hydroxylase (𝜀-CHY), β-lycopene cyclase (β-LCY) and β-carotene hydroxylase (β-CHY) except for phytoene desaturase (PDS) and 𝜀-lycopene cyclase (𝜀-LCY) were systematically up-regulated in leaves of the transgenic *Arabidopsis* plants (**Figure 9**). *GGPS, 𝜀-CHY, β-LCY*, and *β-CHY* exhibited the increased expression levels, but *ZDS, PSY*, and *𝜀-LCY* showed no changes in expression level and *PDS* was down-regulated in the transgenic seeds (**Figure 9**). Under NaCl and drought stresses, the genes encoding the key enzymes in ABA biosynthesis, zeaxanthin epoxidase (ZEP), 9-cis-epoxycarotenoid dioxygenase (NCED), and xanthoxin dehydrogenas (ABA2) were up-regulated, and abiotic stress-responsive genes encoding pyrroline-5-carboxylate synthase (P5CS), SOD, ascorbate peroxidase (APX), and dehydroascorbate reductase (DHAR) were also found to be up-regulated (**Figure 10**).

## Discussion

### *IbARF5* Increases Carotenoid Contents and Salt and Drought Tolerance

In plants, ARFs encode important transcription factors which regulate the expression of genes in response to auxin (Guilfoyle and Hagen, 2007). Several ARF transcription factor genes have been cloned from *Arabidopsis*, rice and tomato, and were found to play crucial roles in plant growth and developmental processes (Harper et al., 2000; Waller et al., 2002; Ellis et al., 2005; Okushima et al., 2005; Wilmoth et al., 2005; Wang et al., 2011; Ren et al., 2017; Liu et al., 2018). However, there is no report on the ARF transcription factors in improving carotenoid contents and abiotic stress tolerance in plants. Previous studies demonstrated that *AtARF5* affected lateral organ development, primary root initiation, flower primordium initiation, and cotyledon development in *Arabidopsis* (Krogan et al., 2012) and *SlARF5* controlled fruit set and development in tomato (Liu et al., 2018). In the present study, the *IbARF5* gene was isolated from sweetpotato line HVB-3 with high carotenoid content. We found that its overexpression significantly increased the content of carotenoids and enhanced the tolerance to salt and drought in transgenic *Arabidopsis* (**Tables 1**, **2** and **Figures 6**, **7**).

### *IbARF5* Up-Regulates the Genes Involved in Carotenoid Biosynthesis

It has been shown that carotenoid biosynthesis is mainly regulated at the transcript level of genes encoding the biosynthetic enzymes (Römer and Fraser, 2005; Sandmann et al., 2006; Li et al., 2017; Kang et al., 2018). In this study, we found that the key genes in carotenoid biosynthesis, *GGPS, ZDS, PSY, 𝜀-CHY, β-LCY*, and *β-CHY* in leaves and *GGPS, 𝜀-CHY, β-LCY*, and *β-CHY* in seeds of transgenic *Arabidopsis* were significantly up-regulated (**Figure 9**), which corresponded with the increase of carotenoid contents in transgenic *Arabidopsis* (**Tables 1** and **2**). These findings suggest that *IbARF5* positively controls the expression of carotenoid biosynthetic genes, which resulted in the increased carotenoid contents in transgenic *Arabidopsis*. Overexpression of the *Orange* gene (*IbOr*) from sweetpotato increased carotenoid accumulation and abiotic stress tolerance in transgenic sweetpotato, potato, and alfalfa (Kim et al., 2013a; Goo et al., 2015; Wang et al., 2015). Furthermore, it was proved that similar to AtOr of *Arabidopsis*, IbOr directly interacted with PSY and increased carotenoid accumulation (Park et al., 2016; Kim et al., 2018). Therefore, the precise underlying mechanisms of *IbARF5* in plant carotenoid accumulation need to be further investigated. In addition, we are developing the *IbARF5*-overexpressing sweetpotato plants for further analyzing its roles in carotenoid accumulation of the storage roots.

### *IbARF5* Up-Regulates the Genes Involved in ABA Biosynthesis

Carotenoids, especially β-branch carotenoids, serve as precursors for ABA biosynthesis and play a crucial role in plant tolerance and adaptation to abiotic stresses (Demmigadams and Adams, 2002; Xiong and Zhu, 2003; Sah et al., 2016). ABA regulates the expression of ABA-dependent stress-responsive genes and the increased level of ABA has been found to enhance the tolerance to salt and drought (Tuteja, 2007; Vishwakarma et al., 2017). It was reported that overexpression of *IbMIPS1, IbZDS*, and *IbLCYB2* increased the level of ABA, which led to the enhanced tolerance to salt and drought in sweetpotato (Zhai et al., 2016; Li et al., 2017; Kang et al., 2018). In this study, the *IbARF5*-overexpressing *Arabidopsis* seeds showed the increased sensitivity to ABA in germination (**Figure 8**). The ABA biosynthetic genes *IbZEP, IbNCED*, and *IbABA2* were up-regulated and ABA level was also significantly increased in transgenic *Arabidopsis* (**Figures 7B** and **10**). These results suggest that overexpression of *IbARF5* confers salt and drought tolerance by up-regulating the ABA biosynthetic genes and increasing ABA level in transgenic *Arabidopsis*.

### *IbARF5* Up-Regulates Abiotic Stress-Responsive Genes and Changes Abiotic Stress-Associated Components

It is reported that the high level of ABA increases the transcript level of *P5CS*, which leads to more accumulation of proline under abiotic stresses (Sripinyowanich et al., 2013). Proline plays a pivotal role in maintaining osmotic balance, protecting integrity membrane and increasing reactive oxygen species (ROS) scavenging capacity, and its elevated level enhances salt and drought tolerance in plants (Yoshiba et al., 1997; Maggio et al., 2002; Neisiani et al., 2009; Gill and Tuteja, 2010; Kang et al., 2018). SOD as the first line of defense against ROS is induced by abiotic stresses to promote ROS scavenging (Wang et al., 2009). In the present study, *P5CS, SOD, APX*, and *DHAR* were up-regulated, proline level and SOD activity were increased and H_2_O_2_ content was decreased in transgenic *Arabidopsis* under salt and drought stresses (**Figure 7B**). Therefore, it is thought that the enhanced tolerance to salt and drought is due to up-regulation of abiotic stress-responsive genes and change of abiotic stress-associated components in transgenic *Arabidopsis*.

## Conclusion

This study reveals, for the first time, that the *IbARF5* gene from sweetpotato is involved in carotenoid biosynthesis and salt and drought tolerance of plants. Its overexpression increased the contents of carotenoids and conferred the tolerance to salt and drought by up-regulating the key genes involved in carotenoid and ABA biosynthesis and abiotic stress responses in transgenic *Arabidopsis*. This study provides a novel *ARF* gene for improving carotenoid contents and salt and drought tolerance of sweetpotato and other plants.

## Author Contributions

QL and CK conceived and designed the experiments. CK and RL performed the experiments. CK and SH analyzed the data. QL, HZ, and NZ contributed reagents, materials, and analysis tools. QL and CK wrote the paper. All authors read and approved the final manuscript.

## Conflict of Interest Statement

The authors declare that the research was conducted in the absence of any commercial or financial relationships that could be construed as a potential conflict of interest.

## Footnotes

1 https://blast.ncbi.nlm.nih.gov/Blast.cgi

2 https://www.ncbi.nlm.nih.gov/orffinder/

3 https://www.ncbi.nlm.nih.gov/sutils/splign/splign.cgi?textpage=online&level=form

## Supplementary Material

FIGURE S1 Sequence alignment of IbARF5 with its homologs from other plants. Characteristic regions of ARF5 are indicated above the IbARF5 sequence. ,Plant-specific B3-DNA binding domain; ,Auxin_resp; ,AUX_IAA.

FIGURE S2 Expression analysis of *IbARF5* in the transgenic *Arabidopsis* plants. The *Arabidopsis actin* gene was used as an internal control. Data are presented as means ± SE (*n* = 3). ^∗∗^ indicates a significant difference from that of WT at *P* < 0.01 by Student’s *t*-test.

TABLE S1 Primers used in this study.
