# Supplementary figures and images for: A Sweetpotato Auxin Response Factor Gene (IbARF5) Is Involved in Carotenoid Biosynthesis and Salt and Drought Tolerance in Transgenic Arabidopsis
Source: Front Plant Sci. 2018 Sep 11;9:1307. doi: 10.3389/fpls.2018.01307 (PMC6141746; doi:10.3389/fpls.2018.01307)

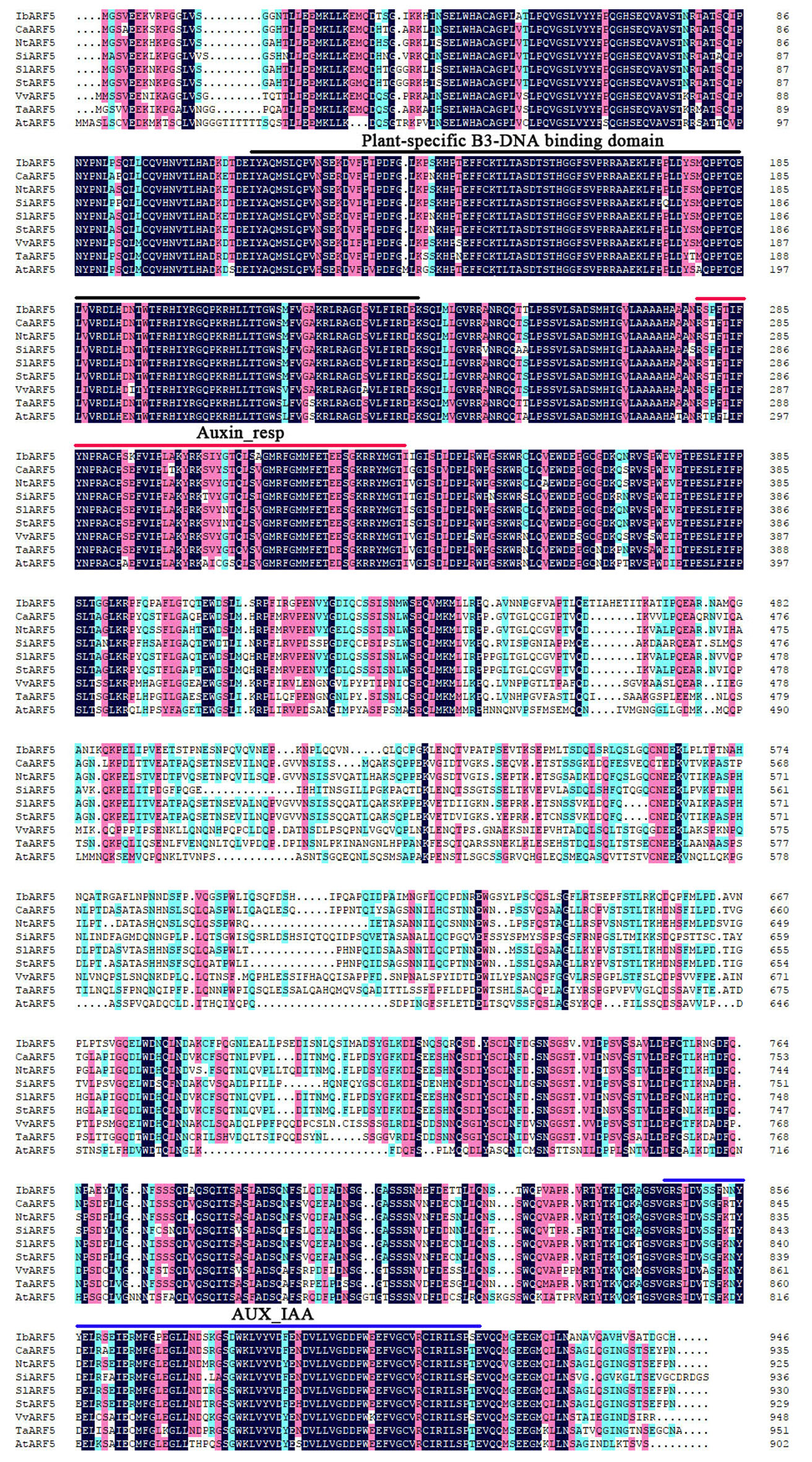

Supplement: FIGURE S1 — Sequence alignment of IbARF5 with its homologs from other plants. Characteristic regions of ARF5 are indicated above the IbARF5 sequence. ,Plant-specific B3-DNA binding domain; ,Auxin_resp; ,AUX_IAA. [file Image_1.JPEG]

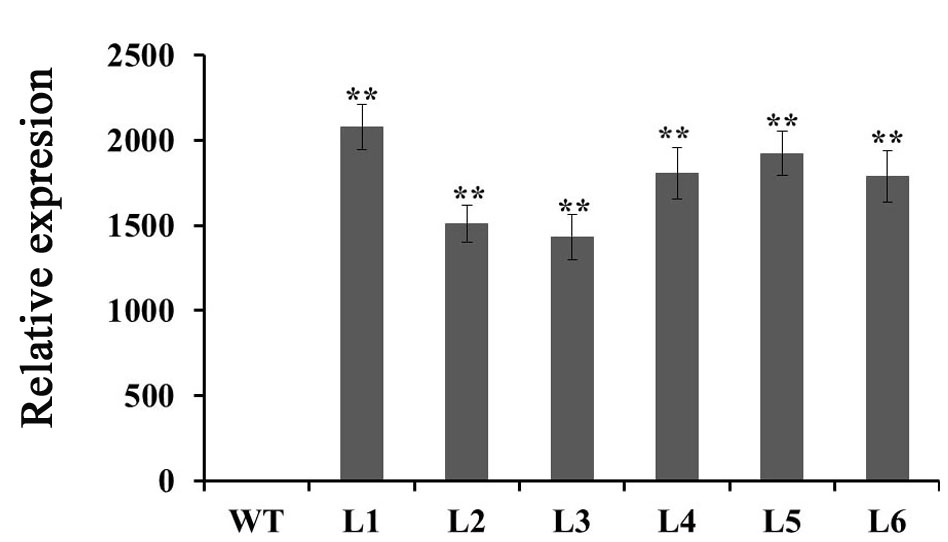

Supplement: FIGURE S2 — Expression analysis of IbARF5 in the transgenic Arabidopsis plants. The Arabidopsis actin gene was used as an internal control. Data are presented as means ± SE (n = 3). ∗∗ indicates a significant difference from that of WT at P < 0.01 by Student’s t-test. [file Image_2.JPEG]
