# Supplementary material for: A Sweetpotato Auxin Response Factor Gene (IbARF5) Is Involved in Carotenoid Biosynthesis and Salt and Drought Tolerance in Transgenic Arabidopsis
Source: Front Plant Sci. 2018 Sep 11;9:1307. doi: 10.3389/fpls.2018.01307 (PMC6141746; doi:10.3389/fpls.2018.01307)
Supplement: TABLE S1 — Primers used in this study. [file Table_1.DOC]

|  |  |  |  |  |  |  |
| --- | --- | --- | --- | --- | --- | --- |

| **Table S1** Primers used in this study | |
| --- | --- |
| Primer name | Primer sequence (5’-3’) |
| Primers for 5’/3’ RACE | |
| 5GSP1 | CTGGCCGAAGTGATCTGTGA |
| 5GSP2 | AAGACCGCACTTGAAACAGA |
| 5GSP3 | ACTTTGTCTTTGGCTCTGGT |
| 3GSP1 | TAAATGATTCCAAAGGCTCG |
| 3GSP2 | AGATGGGCGAGGAAGGAATG |
| Primers for constructing vectors | |
| OS-F- *BamH* I | CGGGATCCATGGGTTCTGTTGAAGAAAAAG |
| OS-R-*Sac*I | CGAGCTCTCAGTGACAACCATCGGTCG |
| 83S-F-*Spe*I | GACTAGTATGGGTTCTGTTGAAGAAAAAG |
| 83S-R-*Asc*I | AGGCGCGCCTCAGTGACAACCATCGGTCG |
| pBD-F-*Nde*I | GGAATTCCATATGATGGGTTCTGTTGAAGAAAAAG |
| pBD-R-*Sal*I | ACGCGTCGACTCAGTGACAACCATCGGTCG |
| pBD-1-F-*Nde*I | GGAATTCCATATGATGGGTTCTGTTGAAGAAAAAG |
| pBD-1-R-*Sal*I | ACGCGTCGACTCACGACTTTTCATCCCTGATAAACA |
| pBD-2-F-*Nde*I | GGAATTCCATATGAGCCCGTTCACGATTTTTTAC |
| pBD-2-R-*Sal*I | ACGCGTCGACCCTGGAGGCAACGCCACTT |
| pBD-3-F-*Nde*I | GGAATTCCATATGGGCGATTTCCAAAATCCCG |
| pBD-3-R-*Sal*I | ACGCGTCGACTCAGTGACAACCATCGGTCG |
| Primers for identifying transformants | |
| 35S-F | GAACTCGCCGTAAAGACTGG |
| *IbARF5*-R | TCAGTGACAACCATCGGTCG |
| Primers for qRT-PCR | |
| *Ibactin*-F | AGCAGCATGAAGATTAAGGTTGTAGCAC |
| *Ibactin*-R | TGGAAAATTAGAAGCACTTCCTGTGAAC |
| *IbARF5*-F | TCCAACCAGAGCCAAAGACA |
| *IbARF5*-R | GTGATCTGTGATTGGGCGTC |
| *Atactin*-F | GCACCCTGTTCTTCTTACCGA |
| *Atactin*-R | AGTAAGGTCACGTCCAGCAAGG |
| *AtGGPS*-F | GCCAATCTGAACCATCCTCT |
| *AtGGPS*-R | ACGGAGAGGAACAGCTGAAT |
| *AtPDS*-F | CGAGATGCTGACATGGCCAGA |
| *AtPDS*-R | GTCGGTCACGCGCTCAGGTA |
| *AtZDS*-F | CCATCGTCACGAGGCCTAGAA |
| *AtZDS*-R | TGTGTATGAACCGGCGAGGA |
| *AtPSY*-F | ATGATCGATGCGGTGAAGTTTGCG |
| *AtPSY*-R | TGAAGCATTTGGCCCATCCACAAG |
| *Atε-LCY*-F | ACCTTAGCTCGAAAGTTGACAG |
| *Atε-LCY*-R | CACCAACTTCGTATTGCAAGAG |
| *Atβ-LCY*-F | AGGATGAACCATTCCTGCTG |
| *Atβ-LCY*-R | ATTGAGGAAGACGAGCGTTG |
| *Atβ-CHY*-F | TGGAAGAACGGAAACAAAGC |
| *Atβ-CHY*-R | AGCTTGTCGTGCTTTCTGGT |
| *Atε-CHY*-F | GGCACGCTTCTCTATGGAATA |
| *Atε-CHY*-R | GAATCCATAAGAGAGGAGAC |
| *AtZEP*-F | CGGAGCTTTCTTCTTGATGG |
| *AtZEP*-R | TCGATTTCGGAGTTTTCCTG |
| *AtNCED*-F | CGCCGGTTTAGTTTATTTCAATGGT |
| *AtNCED*-R | AATCGTACCGACCCGAAGTTTCTAA |
| *AtABA1*-F | TACTTGGGGTAAAGGGCGTG |
| *AtABA1*-R | CCAAGGACCCAGTCAAGCAT |
| *AtP5CS*-F | GCCTGATGCACTTGTTCAGA |
| *AtP5CS*-R | TTGAGCAATTCAGGGACCTC |
| *AtSOD*-F | ATGAGAAGTTCTATGAAGAG |
| *AtSOD*-R | GTCTTTATGTAATCTGGT |
| *AtAPX*-F | CTCTGGGACGATGCCACAAG |
| *AtAPX*-R | CTCGACCAAAGGACGGAAAA |
| *AtDHAR*-F | ATGGTCCTTTTATCGCCGGG |
| *AtDHAR*-R | GCCCATCCAGAGATCACACA |
